# Surviving starvation simply without TFEB

**DOI:** 10.1371/journal.pbio.3000285

**Published:** 2019-05-28

**Authors:** Alexander A. Soukas, Ben Zhou

**Affiliations:** 1 Department of Medicine, Diabetes Unit, and Center for Genomic Medicine, Massachusetts General Hospital, Boston, Massachusetts, United States of America; 2 Department of Medicine, Harvard Medical School, Boston, Massachusetts, United States of America; 3 Broad Institute of Harvard and MIT, Cambridge, Massachusetts, United States of America

## Abstract

Starvation is among the most ancient of selection pressures, driving evolution of a robust arsenal of starvation survival defenses. In order to survive starvation stress, organisms must be able to curtail anabolic processes during starvation and judiciously activate catabolic pathways. Although the activation of metabolic defenses in response to nutrient deprivation is an obvious component of starvation survival, less appreciated is the importance of the ability to recover from starvation upon re-exposure to nutrients. In order for organisms to successfully recover from starvation, cells must be kept in a state of ready so that upon the return of nutrients, activities such as growth and reproduction can be resumed. Critical to this state of ready is the lysosome, an organelle that provides essential signals of nutrient sufficiency to cell growth-activating pathways in the fed state. In this issue, Murphy and colleagues provide evidence that exposure of *Caenorhabditis elegans* roundworms to 2 simple nutrients, glucose and the polyunsaturated fatty acid linoleate, is able to render lysosomal function competent to activate key downstream starvation recovery pathways, bypassing the need for a master transcriptional regulator of lysosomes. These findings provide a quantum leap forward in our understanding of the cellular determinants that permit organisms to survive cycles of feast and famine.

## Abstract 2

Organisms require elaborate systems to defend against nutrient stress. This Primer explores recent evidence that the transcription factor TFEB, a master regulator of starvation defences, also primes animals for recovery from starvation once food becomes available.

## Introduction

It is rare for organisms to encounter constant food supply in the wild. More commonly, animals are exposed to extended periods where food is scarce and therefore must execute metabolic defenses to survive the stress of nutrient deprivation. Similarly, organisms must be able to rapidly respond when nutrients become available to resume growth, development, and reproduction. Thus, efficient and rapid response to changing nutrient conditions is critical for survival of the organism and the species as a whole.

Organisms have evolved elaborate nutrient sensing pathways that permit adaptation to changing environmental conditions. At the cellular level, distinct pathways that detect intracellular and extracellular levels of sugars, lipids, amino acids, and other nutrients govern responses to food abundance [1]. In animal models, many of these same pathways coordinate organism-level responses to altered nutrient levels [2]. In mammals, starvation leads to reductions in nutrient levels, corresponding to drops in anabolic hormones such as insulin and increases in hormones that are protective against reductions in blood glucose such as glucagon, cortisol, and epinephrine. Nutrients and hormones alike are integrated at the cellular level, governing the balance of anabolic and catabolic activities. Organisms need to be prepared to rapidly switch between these activities with alterations in feeding status. Fundamental understanding of the cellular organelles and genetic pathways mediating rapid responses to shifts in nutrient levels and signaling hormones is beginning to emerge.

*Caenorhabditis elegans* shares key nutrient sensing and response pathways with mammals, making it an outstanding system for studying responses to starvation and feeding. A study of acute starvation in *C*. *elegans* determined that multiple genetic pathways are involved in inhibition of anabolic pathways, including DNA, RNA, and protein synthesis and in activation of catabolic pathways including autophagy and lipolysis [3]. Multiple factors have similarly been identified that are critical for activation of defenses against long-term starvation, including dauer formation affected family member-16/Forhkead box O (DAF-16/FOXO), AMP-activated protein kinase (AMPK), Skinhead transcription factor-1/ Nuclear factor erythroid 2-related factor 2 (SKN-1/NRF2), defective PHAryngeal development family member 4/Forkhead box A (PHA-4/FOXA), helix-loop-helix family member 30/transcription factor EB (HLH-30/TFEB), and the tumor suppressor retinoblastoma (Rb) [4–8]. In spite of this fairly granular understanding of genetic pathways involved in the response to starvation, detailed molecular mechanisms governing recovery from starvation remain incompletely characterized.

Previous studies have identified AMPK and mechanistic target of rapamycin complex 1 (mTORC1) as cellular, nutrient-sensing protein kinases that respond antagonistically to alterations in cellular energy and nutrient levels under starvation and feeding [9]. Generally speaking, mTORC1 is a nutrient sufficiency factor, activated by growth factors, insulin, and nutrients, that stimulates anabolic cellular pathways [10]. Alternatively, AMPK is activated upon energy deficiency, stimulating catabolic processes to restore cellular adenosine triphosphate (ATP) levels [11]. Thus, during starvation, AMPK is activated and mTORC1 inhibited, increasing catabolic processes such as autophagy and lipolysis and suppressing anabolic activities such as protein synthesis and lipogenesis [11]. With nutrient sufficiency, AMPK activity is suppressed and mTORC1 stimulated, increasing anabolism and decreasing catabolism. This balance is critical to responses to changing nutrient levels, because animals deficient in AMPK and mTORC1 show defective responses to starvation and feeding.

Both mTORC1 and AMPK can be activated at the lysosome [12, 13], an acidified, membrane-bound organelle containing hydrolytic enzymes that break down many kinds of biomolecules. Besides its function in degradation for cellular recycling, emerging evidence demonstrates that lysosomes are a central hub for sensing nutrients and metabolic adaption [14]. In these organelles, ion and nutrient transporters, protein kinases and phosphatases, and transcription factors and transcriptional regulators integrate important cellular parameters such as nutrient abundance, energy levels, and cellular stressors and translate them into instructions that steer cellular metabolism towards anabolism and proliferation versus catabolism and quiescence [15].

It is well established that mTORC1 is recruited to and activated on lysosomes in the presence of both growth factors and nutrients. In addition to sensing cytosolic amino acids, mTORC1 senses a lysosomal pool of amino acids via Ras-related GTP-binding protein (Rag)-interacting and Ragulator-interacting transmembrane proteins, including the vacuolar H^+^-ATPase (VHA) proteins and a lysosomal transmembrane transporter solute carrier family member 38A9 (SLC38A9) [16–18]. Recent findings similarly indicate that glucose and cholesterol also promote Rag-dependent mTORC1 activation at the lysosome [12, 19]. How these nutrients are integrated upstream of the Rags and whether other nutrients like fatty acids similarly activate mTORC1 remain poorly understood.

Crosstalk between the lysosome and nucleus is mainly controlled by the master transcriptional regulator TFEB, along with other members of the microphthalmia (MiT) basic helix-loop-helix–leucine-zipper (bHLH-Zip) transcription factor E (TFE) family [20, 21]. TFEB plays a pivotal role in the activation of catabolic cellular processes, such as lysosomal biogenesis, lipolysis, fatty acid oxidation, and autophagy [7]. TFEB activity is regulated through multiple mechanisms including post-translational modifications, protein–protein interactions, and spatial localization. Under nutrient-rich conditions, TFEB is recruited to the surface of lysosome via its interaction with Rag GTPases and is phosphorylated and inactivated by mTORC1 and other protein kinases [22]. Under nutrient deprivation stress, TFEB is rapidly dephosphorylated and shuttles into the nucleus, activating a set of stress defense genes, including lysosomal hydrolases, pumps, permeases, and autophagic regulatory genes [23]. There is feedback regulation from TFEB to mTORC1, as TFEB both increases lysosomal biogenesis and transcriptionally increases Rag family member D (RagD) expression, thereby promoting activation of mTORC1 signaling [24]. HLH-30, which is the ancient ancestor of mammalian TFEB/TFE3 in *C*. *elegans*, has also been shown to be a starvation-responsive master regulator of lysosome biogenesis and autophagy in the worm [7, 25, 26].

**Fig 1:**
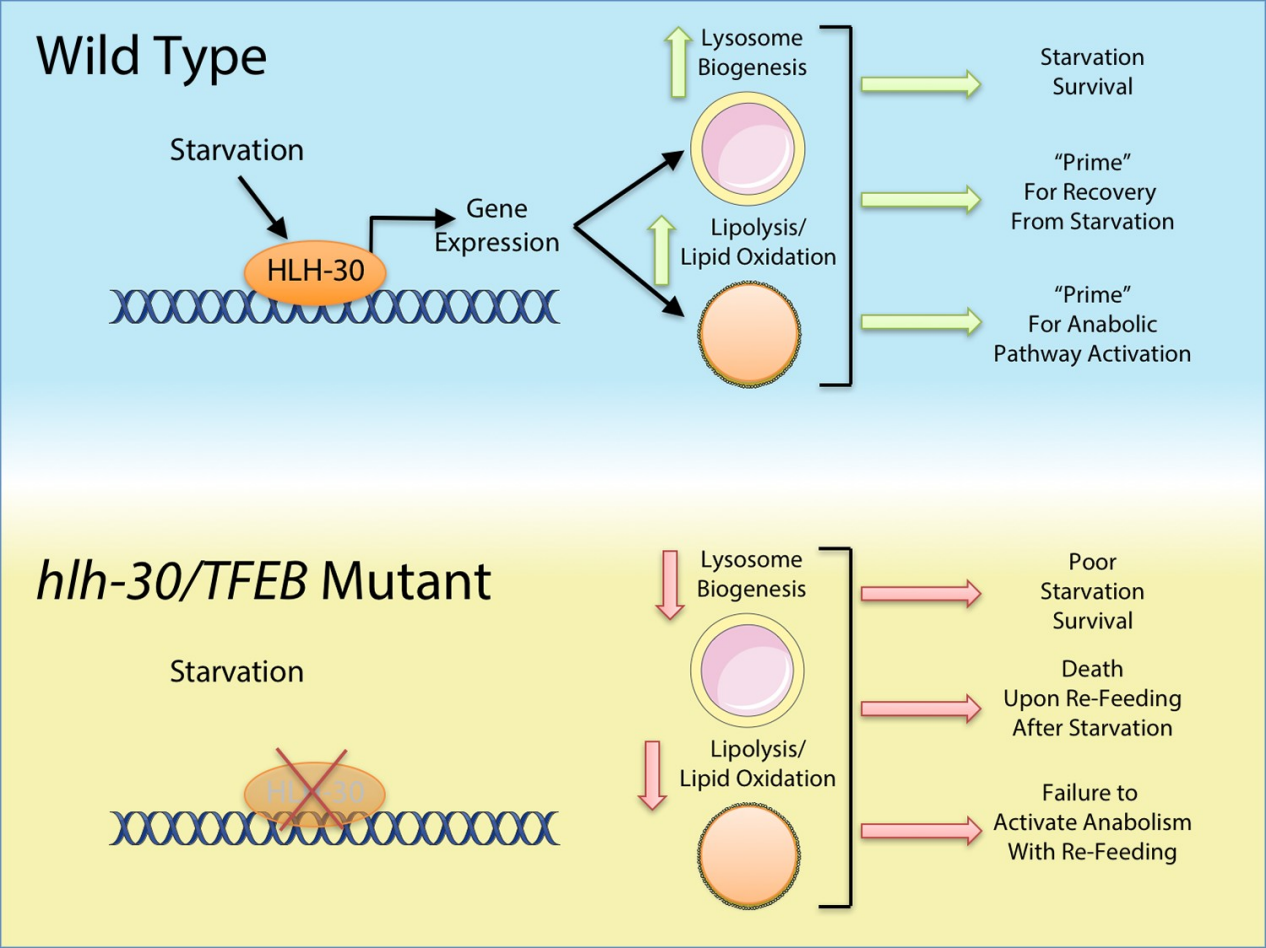
HLH-30/TFEB regulates a complex transcriptional network critical for nutrient sensing and starvation defenses. In wild-type *C*. *elegans* under starvation conditions, HLH-30 activates lysosome biogenesis and catabolic pathways, which encourage starvation survival and prime the animal for recovery from starvation upon re-feeding. Without HLH-30, *C*. *elegans* fail to induce lipolytic and lysosomal genes such as *lipl-2* and VHA genes, leading to diminished starvation survival and failure to recover from starvation once food is reintroduced. Murphy and colleagues in this edition of *PLOS Biology* report that simple nutrients glucose and linoleic acid can bypass the need for HLH-30 in recovery from starvation. It remains unknown how glucose and linoleic acid bypass the need for HLH-30 but at least in part it may be through restoration of lysosomal function and mTORC1 activity downstream of HLH-30 action. HLH-30, helix-loop-helix family member 30; mTORC1, mechanistic target of rapamycin complex 1; TFEB, transcription factor EB; VHA, vacuolar H^+^-ATPase.

A report in the current edition of *PLOS Biology* shows that the *C*. *elegans* ancestral gene for the MiT/TFE transcription factor family *hlh-30* is required to couple lysosomal nutrient sensing to survival during nutrient stress, probably by providing critical metabolites required for recovery from breakdown of complex substrates in lysosomes [27]. Previous studies have demonstrated that *C*. *elegans* lacking *hlh-30* die prematurely under starvation [3, 18], underscoring the importance of *hlh-30* for the physiological response to starvation. Furthering these findings, Murphy and colleagues find that *hlh-30* is not only required for starvation survival but also for recovery following re-feeding the bacterial food source *Escherichia coli* after starvation (Fig 1). *C*. *elegans* mutants lacking *hlh-30* do not survive re-feeding with *E*. *coli* following starvation, indicating critical roles for *hlh-30* both in sustaining survival during starvation and in recovery following re-feeding. Kyoto encyclopedia of genes and genomes (KEGG) pathway analysis of transcriptomewide RNA sequencing (RNAseq) data demonstrate that starved *hlh-30* mutant worms displayed altered levels of gene transcripts encoding proteins involved in amino acid metabolism, fatty acid metabolism, and lysosomal function compared with their wild-type counterparts. These interesting findings suggest first that *hlh-30* is necessary for metabolic adaptation to starvation, and second that compensatory alterations may occur in attempts to resist starvation stress in *hlh-30* mutants.

The authors find that pre-exposure of *hlh-30* mutants to *C*. *elegans* maintenance medium (CeMM), a fully-defined chemical medium that contains various simple nutrients, is able to nearly completely bypass the requirement for HLH-30 for survival upon re-feeding following starvation. Pre-exposure to CeMM restores tricarboxylic acid (TCA) cycle and linoleic acid metabolites in starved *hlh-30* mutant worms, probably at least partially due to increased expression of a lysosome lipase *lipl-2*. *lipl-2* expression, which fails to increase in starved *hlh-30* mutants, is necessary for both starvation resistance and recovery upon re-feeding. Through metabolomic analysis and add-back experiments, the authors determine that the 2 active components of CeMM that are necessary and sufficient for *hlh-30* mutants to survive re-feeding with *E*. *coli* following starvation are glucose and the polyunsaturated fatty acid linoleic acid. CeMM increases the transcription of lysosomal vacuolar H^+^-ATPase (VHA) proton pump genes even in the absence of HLH-30, thereby restoring lysosome acidification and number. Thus, even in the absence of functional HLH-30, the functional components in CeMM (glucose and linoleic acid) prepares animals for re-feeding following starvation by enabling lysosomal function.

These findings are very important for 4 reasons. First, they demonstrate that TFEB/HLH-30 is critical not only for survival under starvation but also for “priming” the animal for recovery upon re-feeding. The authors conclusively demonstrate that these functions of HLH-30 are dependent on normal lysosome function. Second, simple metabolites such as glucose and polyunsaturated fatty acids can bypass the need for TFEB/HLH-30 by activating the transcription of genes involved in the TCA cycle, lipolysis, as well as lysosome biogenesis, promoting stress defenses upon re-feeding following starvation. Third, the authors go on to demonstrate that at least one mechanism by which simple metabolites in CeMM prevent lethality upon re-feeding following starvation in TFEB/*hlh-30* mutants is through up-regulation of the small GTPase *ragc-1*, an important component and activator in the mTORC1 signaling pathway. Although more work is necessary to definitively connect these findings to mTORC1, the data presented decisively show that inhibition of mTORC1 prevents recovery from starvation upon re-feeding. Finally, these findings further prove the central role of lysosome in mediating organismal survival upon recovery from starvation stress, not only because of its catabolic functions that provide nutrients but also in generating certain metabolites as survival signals.

It is still unclear how glucose and linoleic acid coordinately activate lysosome biogenesis and gene expression bypassing TFEB/HLH-30. The authors found that overexpression of lipase *lipl-2*, which acts genetically downstream of *hlh-30*, is not sufficient alone to rescue starvation lethality but is capable of some rescue when supplied with glucose. Thus, lysosomal lipolysis is essential but not sufficient for surviving starvation and recovery upon re-feeding. A possibility is that mannose-6-phosphate glycosylation of certain enzymes is essential for sorting, trafficking, and localization in the lysosome. Mannose-6-phosphate can be derived from fructose-6-phosphate, an intermediate of glycolysis. Thus, glucose may provide essential substrates from glycolytic pathway for glycosylation to maintain lysosomal function. In line with this, the authors found that glycolytic intermediates are increased in *hlh-30* worms supplemented with CeMM. Previous studies from our lab and others have shown that AMPK is critical for starvation survival [8], and AMPK can sense glucose indirectly through AMP levels and through fructose-1,6-bisphosphate and aldolase on the lysosome [28]. It remains to be demonstrated whether CeMM activates mTORC1 through modulation of AMPK activity. Finally, it remains a distinct possibility that other transcription factors including DAF-16 and PHA-4 may contribute to the up-regulation of lysosome biogenesis genes in the absence of functional TFEB/HLH-30.

Another interesting finding in this study is that decreased numbers of autophagosomes are restored by CeMM supplementation in *hlh-30* mutant worms, indicating that simple metabolites can directly activate autophagosome formation downstream of HLH-30. Consistent with this finding, a previous study demonstrated that polyunsaturated fatty acids liberated by starvation-induced lipolysis stimulate Beclin-independent autophagy, promoting starvation defenses [29]. Although the authors demonstrate that the autophagosome formation gene Beclin1/*bec-1* is not required for the beneficial effect of glucose and linoleic supplementation in starved *hlh-30* mutant worms, it is still unclear whether autophagy is involved in this process.

Because defective lysosome dysfunction is associated with many aging-related diseases such as Parkinson’s disease and Alzheimer’s disease, as well as with a decline in lifespan, targeting lysosomal functional capacity is emerging as a potential strategy to promote longevity and prevent aging-associated diseases. Overexpression of *hlh-30* extends lifespan by 15% to approximately 20% versus wild-type worms [25], and similarly, overexpression of lysosome lipase *lipl-1* or *lipl-3* also extends *C*. *elegans* life span [26]. Given the important role of HLH-30 in promoting lipolysis, activation of HLH-30 may extend lifespan through increased lipolysis and specifically, certain lipid metabolites. In line with this, the ω-6 fatty acid di-homo-γ-linoleic acid (DGLA), increases *C*. *elegans* life span through the activation of autophagy in well-fed conditions [29]. It remains a tantalizing possibility, as substantiated by this study by Murphy and colleagues, that provision of simple nutrients such as glucose and linoleic acid may improve lysosomal function in aging and disease, thereby promoting healthy aging and mitigating devastating diseases such as neurodegeneration and lysosomal storage diseases.

## Abbreviations

AMPK: AMP-activated protein kinase
ATP: adenosine triphosphate
bHLH-Zip: basic helix-loop-helix-leucine-zipper
CeMM: *C*. *elegans* maintenance medium
DAF-16: dauer formation abnormal family member 16
FOXA: Forkhead box protein A
FOXO: Forkhead box protein O
HLH-30: Helix Loop Helix family member 30
KEGG: Kyoto Encyclopedia of Genes and Genomes
MiT: microphthalmia
mTORC1: mechanistic target of rapamycin complex 1
NRF2: Nuclear factor erythroid 2-related factor 2
PHA-4: defective PHAryngeal development family member 4
Rag: Ras-related GTP-binding protein
RagD: Rag family member D
RNAseq: RNA sequencing
SKN-1: SKiNhead transcription factor family member 1
SLC38A9: solute carrier family member 38A9
TCA: tricarboxylic acid
TFE: transcription factor E
TFEB: transcription factor EB
